# 

**Published:** 1894-08

**Authors:** 


					﻿THE
Southern Medical Record.
A Monthly Journal of Medicine and Surgery.
Vol. XXIV. ATLANTA, GA., AUGUST, 1894.	No. 8?
EDITORS :
A. W. GRIGGS, M. D.
j. McFadden gastón, m. d.
WILLIS F. WESTMORELAND, M. D.
LOUIS H. JONES, M. D.
DAN. H. HOWELL, M. D., Business Manager.
Entered at the Postofiice at Atlanta, Ga., as Second-Class Matter.
Tne Franklin Printing and Publishing Co., Atlanta, Ga.
				

